# Photoresponsiveness affects life history traits but not oxidative status in a seasonal rodent

**DOI:** 10.1186/s12983-019-0311-3

**Published:** 2019-04-18

**Authors:** Anna S. Przybylska, Michał S. Wojciechowski, Małgorzata Jefimow

**Affiliations:** 1 Department of Vertebrate Zoology, Nicolaus Copernicus University, ul. Lwowska 1, 87-100 Toruń, Poland; 2 Department of Animal Physiology, Nicolaus Copernicus University, ul. Lwowska 1, 87-100 Toruń, Poland

**Keywords:** Photoresponsiveness, Polymorphism, Reproduction, Life history traits, Basal metabolic rate, Oxidative stress

## Abstract

**Background:**

Shortening photoperiod triggers seasonal adjustments like cessation of reproduction, molting and heterothermy. However there is a considerable among-individual variation in photoresponsiveness within one population. Although seasonal adjustments are considered beneficial to winter survival, and natural selection should favor the individuals responding to changes in photoperiod (responders), the phenotype non-responding to changes in day length is maintained in population. Assuming the same resource availability for both phenotypes which differ in strategy of winter survival, we hypothesized that they should differ in life history traits. To test this we compared reproductive traits of two extreme phenotypes of Siberian hamster *Phodopus sungorus* – responding and non-responding to seasonal changes in photoperiod. We bred individuals of the same phenotype and measured time to first parturition, time interval between litters, offspring body mass 3, 10 and 18 days after birth and their growth rate. We also analyzed nest-building behavior. Additionally, we estimated the correlation between reproduction, and basal metabolic rate (BMR) and oxidative status in both phenotypes to infer about the effect of reproductive output on future investments in somatic maintenance.

**Results:**

Prior to reproduction responding individuals were smaller than non-responding ones, but this difference disappeared after reproduction. Responding pairs commenced breeding later than non-responding ones but there was no difference in time interval between consecutive litters. Responders delivered smaller offspring than non-responders and more out of responding individuals built the nest during winter than non-responding ones. Reproduction did not affect future investments in somatic maintenance. Phenotypes did not differ in BMR and oxidative status after reproduction. However, concentration of reactive oxygen metabolites (ROM) was highest in responding males, and biological antioxidant potential (BAP) was higher in males of both phenotypes than in females.

**Conclusions:**

Delayed breeding in responding Siberian hamsters and high ROM concentration in male responders support our hypothesis that differences in adjustment to winter result in different life history characteristics which may explain coexistence of both phenotypes in a population. We propose that polymorphism in photoresponsiveness may be beneficial in stochastic environment, where environmental conditions differ between winters. We suggest that non-responding phenotype may be particularly beneficial during mild winter, whereas responders would be favored under harsh conditions. Therefore, none of the phenotypes is impaired when compared to the other.

## Background

For small Temperate-Zone endothermic animals winter is one of the most energy demanding periods of the year. Low ambient temperature increases energy requirements, whereas food availability is greatly reduced [1, 2]. To survive animals must adjust their physiology and behavior to prevailing environmental conditions. In small mammals these adjustments include gonadal regression and cessation of reproduction, decrease of body mass (*m*_b_), and molt to a winter coat, all to ensure better thermal insulation and lower energy expenditure. Moreover, heterothermic species use torpor which grants additional energy savings [3–5]. Above changes in physiology and behavior are triggered by shortening day [6–8], and ultimately increase the probability to survive winter and reproduce in spring. However, there is a considerable variation in photoresponsiveness among individuals of the same species, or even within the same population [9, 10]. Some individuals do not respond to changes in photoperiod (non-responding phenotype or non-responders), do not regress gonads, do not enter torpor, and do not change *m*_b_, what may lead to higher energy expenditure and eventually to higher costs of winter survival [6, 8, 11, 12]. The proportion of non-responding individuals varies between species and may reach up to 80% in Prairie voles *Microtus ochrogaster* [13], 47% in Turkish hamsters *Mesocricetus brandti* [14], 50% in white footed mice *Peromyscus leucopus* [15] and 25% in deer mice *Peromyscus maniculatus* [16]. Despite the lack of direct evidence for higher survival of responding individuals (responders), previous studies showed that both, daily and seasonal torpor as well as reduction of normothermic body temperature may increase the life span by decreasing risk of predation [17, 18], reducing the overall cost of survival under challenging environmental conditions [17–19] or by reducing the rate of senescence [20–24]. Moreover, cessing reproduction in winter delays reproductive ageing by deceleration of the attrition of ovarian follicles [25], increase of litter size and decrease of the number of failed pregnancies [26]. If seasonal adjustments in physiology (including heterothermy) and behavior are considered beneficial to winter survival and reproductive success, natural selection should favor responding phenotype (responders), eventually leading to elimination of the non-responding one. Nevertheless, both phenotypes are maintained in populations [10, 27]. Place and Cruickshank [25] suggested, that individuals which respond to short photoperiod should have higher reproductive success than non-responding ones. However, to the best of our knowledge this prediction has not been experimentally verified. We hypothesized that winter phenotypes which differ in their strategy of managing energy resources, should also differ in life history traits. In line with that we predicted that different winter phenotypes are associated with different characteristics of life history traits, and that responding individuals commence reproduction later and deliver smaller litters with bigger offspring than non-responding ones. To verify it, and ultimately to test our hypothesis, we used Siberian hamster *Phodopus sungorus* which is highly seasonal rodent, but with a considerable among-individual variation in photoresponsiveness. On average, depending on a population, between 20 and 60% of individuals do not respond to short photoperiod and remain in their summer status throughout the year [28–32]. We used hamsters that were kept in pairs composed of individuals of the same phenotype (responders or non-responders) and measured time to first parturition, *m*_b_ of offspring and their growth rate in two consecutive litters. Additionally, we analyzed nest-building behavior as a trait that may differ between responders and non-responders, and which may be associated with differences in life history traits.

To inquire about the consequences of reproduction on future somatic maintenance, we analyzed parameters of oxidative stress and antioxidant defense in post-reproductive males and females. Imbalance between reactive oxygen species production and antioxidant defense leads to oxidative stress [33–36], which reflects the cost of somatic maintenance [37]. Since reproduction increases energy expenditure, it was hypothesized that it may lead to increased production of reactive oxygen species and eventually launch the protective mechanisms that limit oxidative damage [38–40]. High costs of antioxidant defense may in turn reduce further investment into somatic maintenance leading to oxidative stress [38, 41, 42]. However, available data on oxidative cost of reproduction are ambiguous, or even contradictory. On the one hand, it was reported that reproductive animals had increased oxidative stress (OS) [38, 43] and decreased antioxidant capacity (AC) [41, 42, 44, 45]. On the other hand, pregnancy and lactation was found to protect against OS [40, 46–48]. Other studies showed negative correlation between life history traits such as litter size or litter mass and OS [38, 49], and positive correlation between clutch size and AC [50]. Assuming that the type of response to winter may affect life history traits and therefore investment into reproduction, we predicted that responders and non-responders differ in their investment in somatic maintenance what would be reflected in different oxidative status after reproduction. Because in most mammals cost of reproduction for males is restricted to mate competition [51, 52], and in Siberian hamsters sire presence in the nest does not affect pup survival and development [53, 54], we also predicted that dams, which bear most of the reproduction cost, invest less into somatic maintenance after reproduction and may be a subject to oxidative stress. To test these predictions, we measured basal metabolic rate (BMR), OS and AC in reproductive and non-reproductive individuals of both phenotypes.

## Results

### Life history traits in two phenotypes of Siberian hamster

All hamsters gained body mass during breeding season (LME: F_(1, 103.59)_ = 69.99, *P* < 0.001), and *m*_b_ of males and females increased by 10 and 17%, respectively (Table 1). Responders differed in *m*_b_ from non-responders only before breeding (LME: F_(1, 103.59)_ = 15.37, *P* < 0.001), when responding individuals were smaller than non-responding ones (Table 1). There was also a significant interaction between reproductive status and sex (LME: F_(1, 111.70)_ = 18.20, *P* < 0.001) and reproducing females weighted more than non-reproducing ones, while in males this relation was opposite (Table 2).

**Table 1:** Comparison of life-history traits between winter phenotypes of Siberian hamster

		Phenotype	
Life history trait		responding	non-responding
*m*_b_ before breeding (g)	males	26.81 ± 3.74	32.66 ± 3.56 ^a^
	females	23.04 ± 2.11	24.97 ± 2.61 ^a^
*m*_b_ after breeding (g)	males	32.79 ± 3.37	34.13 ± 3.62
	females	30.02 ± 4.30	28.22 ± 3.32
litter size	1st	5.33 ± 1.61	4.20 ± 2.04
	2nd	5.11 ± 2.20	4.60 ± 1.51
offspring *m*_b_ (g)	3 days	2.56 ± 0.31	2.94 ± 0.40 ^a^
	10 days	6.43 ± 0.82	6.97 ± 0.92 ^a^
	18 days	12.65 ± 1.85	13.45 ± 1.79 ^a^
growth rate (g day^− 1^)	1st	0.65 ± 0.12	0.69 ± 0.11
	2nd	0.71 ± 0.11	0.71 ± 0.11
time of commencing breeding (days)		36.50 ± 3.56	22.00 ± 2.24 ^a^
time interval between consecutive litters (days)		27.00 ± 5.28	38.50 ± 4.92
degree of use of paper tube %	winter	87.10 ± 12.04	77.78 ± 16.49 ^a^
	summer	66.74 ± 20.24 ^b^	67.67 ± 24.72 ^b^
propensity to build the nest %	winter	91.30	72.50 ^a^
	summer	30.40 ^b^	43.10 ^b^

Values are mean ± SD or median ± SE in case of time of commencement to breeding and time interval between consecutive litters

^a^ difference between phenotypes; *P* ≤ 0.05

^b^ difference between seasons within phenotype; *P* ≤ 0.05

**Table 2:** Comparison of life-history traits between breeding and non-breeding Siberian hamsters

		Phenotype	
Life history trait		breeding	non-breeding
*m*_b_ before breeding (g)	males	30.17 ± 4.54	31.95 ± 4.36
	females	24.72 ± 2.71	23.95 ± 2.44
*m*_b_ after breeding (g)	males	32.37 ± 2.62	35.87 ± 3.87 ^a^
	females	30.12 ± 3.42	26.65 ± 3.03 ^a^
degree of use of paper tube %	winter	84.20 ± 12.81	74.55 ± 18.57
	summer	68.82 ± 20.86	64.02 ± 27.14
propensity to build the nest %	winter	88.3	61.1 ^a^
	summer	43.6 ^b^	31.5 ^b^

Values are mean ± SD

^a^ difference between reproductive status; *P* ≤ 0.05

^b^ difference between seasons within reproductive status; *P* ≤ 0.05

Litter size did not differ between consecutive litters (LME: F_(1, 38)_ = 0.02, *P* = 0.88; Table 1) and it ranged between one and eight pups. Also phenotypes did not differ in litter size (LME: F_(1, 38)_ = 2.18, *P* = 0.15; Table 1).

**Fig. 1:**
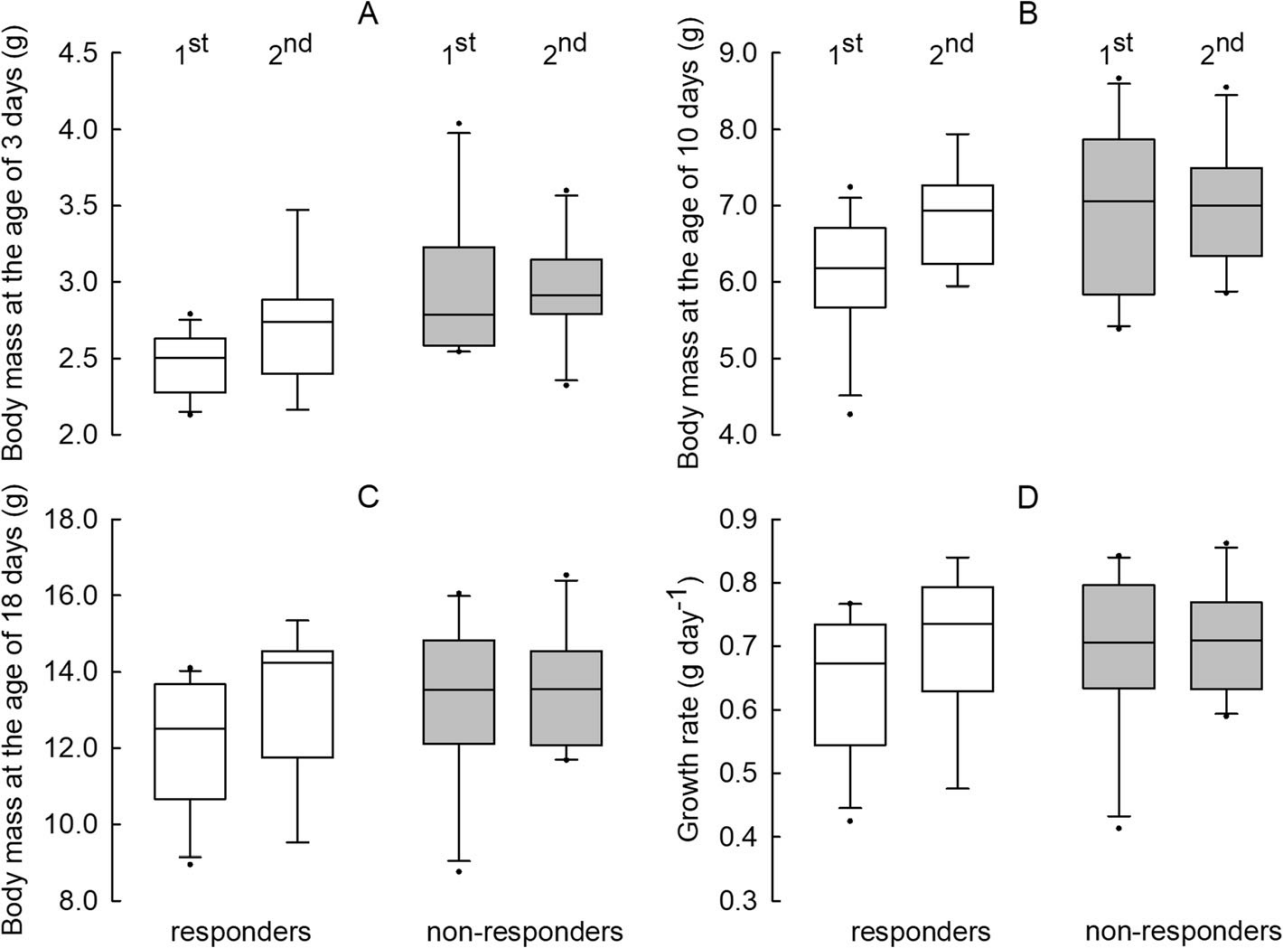
Life history traits (**a**: body mass at the age of 3 days; **b**: body mass at the age of 10 days; **c**: body mass at the age of 18 days; **d**: mean daily growth rate between 3^rd^ and 18^th^ day of life) in responding and non-responding pairs. Ordinal numbers above figures indicate consecutive litters. Lines inside boxes indicate median, while boxes cover the 25th to 75th percentiles. Whiskers indicate maximum value below upper fence and minimum value above lower fence. Dots indicate outliers

Body mass of individual offspring was negatively related to litter size (LME: F_(1, 44.84)_ = 4.68, *P* = 0.04). Independent of the parental phenotype, mean *m*_b_ of individual pup increased with consecutive litters (LME: F_(1, 107.41)_ = 4.11, *P* = 0.04). Offspring *m*_b_ increased with age (LME: F_(1, 93.54)_ = 2156.80, *P* < 0.001), but responding pairs delivered smaller offspring than non-responding ones (LME: F_(1, 48.46)_ = 5.97, *P* = 0.002; Table 1, Fig. 1 a-c). The growth rate did not differ between phenotypes (LME: F_(1, 93.54)_ = 0.84, *P* = 0.36) resulting in smaller *m*_b_ at weaning in offspring of responders (Fig. 1 d).

**Fig. 2:**
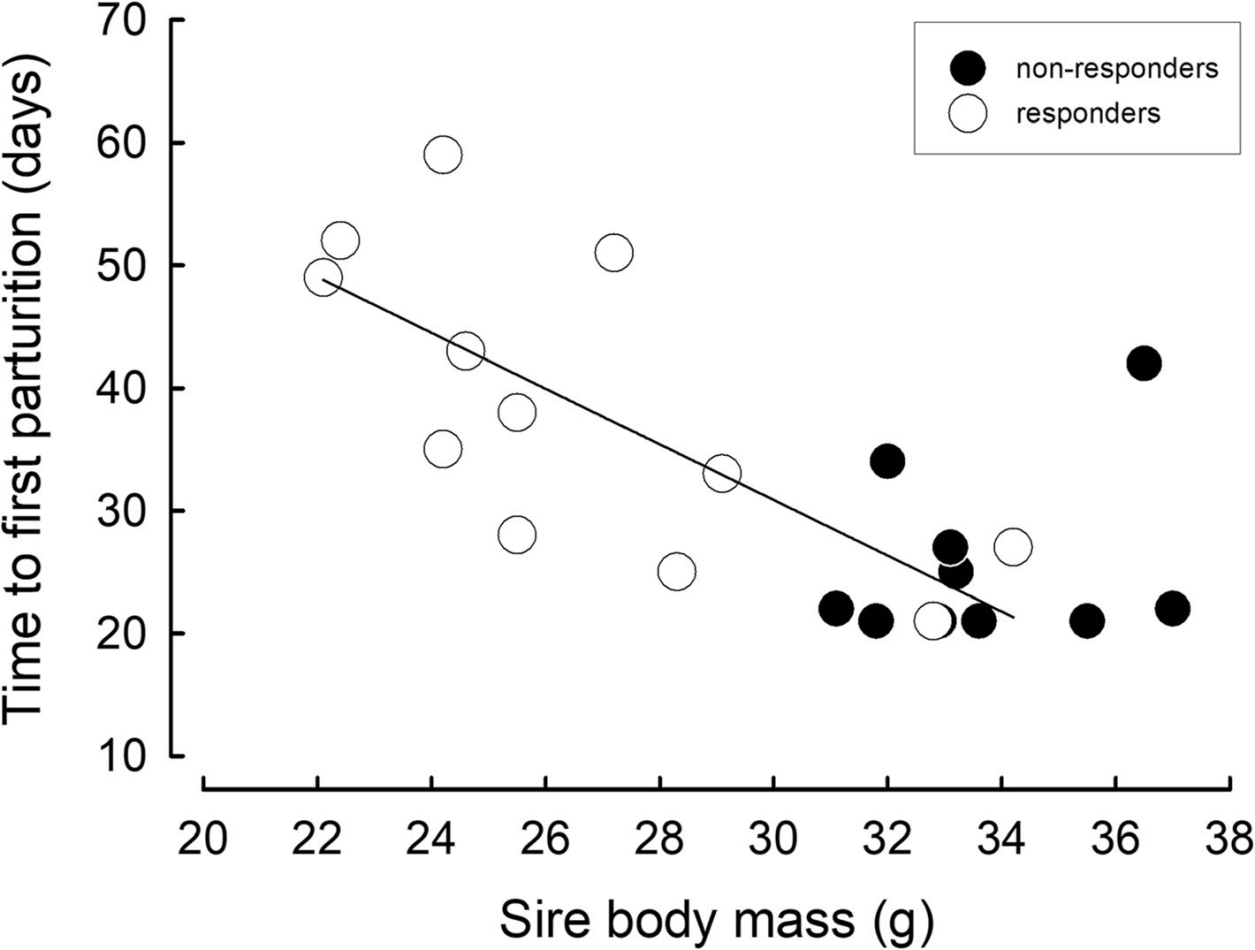
Relationship between sire body mass and time to first parturition in responding and non-responding hamsters. Regression line (y = -2.27 x + 99.00, r^2^ = 0.50, F_(1, 10)_ = 10.03, *P* = 0.01) indicates relationship between sire body mass and time to the first parturition in responding hamsters

Phenotypes differed in the time of commencing breeding (U = 20.00, *P* = 0.008) but not in the time interval between consecutive litters (U = 20.00, *P* = 0.12; Table 1). In both phenotypes dam *m*_b_ correlated neither with the time of first parturition (NR: *r* = − 0.65, N = 8, *P* = 0.08; R: *r* = 0.31, N = 9, *P* = 0.41) nor with the time interval between litters (NR: *r* = 0.02, N = 8, *P* = 0.97; R: *r* = − 0.28, N = 9, *P* = 0.47). However, among responders, bigger sires bred earlier than smaller ones (*r* = − 0.71, N = 12, *P* = 0.01) whereas there was no such relationship among non-responders (*r* = 0.26, N = 10, *P* = 0.47; Fig. 2). There was also no correlation between sire *m*_b_ and time interval between consecutive litters (NR: *r* = − 0.17, N = 8, *P* = 0.69; R: *r* = − 0.21, N = 9, *P* = 0.58).

### Nest-building behavior

Both the proportion of using the paper tube as well as the propensity to build the nest were repeatable (*r* = 0.46, 95% CI 0.40–0.52, *P* < 0.001; and *r* = 0.25, 95% CI 0.10–0.38, *P* = 0.05; respectively). In winter both phenotypes increased the use of paper tube (LME: F_(1, 218)_ = 83.10, *P* < 0.001) and the propensity to build the nest (χ^2^_(1, 296)_ = 46.91, *P* < 0.001), however, in responding individuals this increase was greater than in non-responding ones (degree of using paper tube: LME: F_(1, 218)_ = 10.61, *P* = 0.001; propensity to build the nest: χ^2^_(1, 148)_ = 6.58, *P* = 0.01, Table 1). Non-breeding individuals tended to use paper tubes slightly less than breeding ones (LME: F_(1, 70)_ = 2.99, *P* = 0.09). Breeding and non-breeding hamsters differed in their propensity to build the nest only in winter (χ^2^_(1, 148)_ = 14.96, *P* < 0.001), while in summer, the percentage of hamsters building the nest was similar (Table 2).

### BMR and oxidative status

**Fig. 3:**
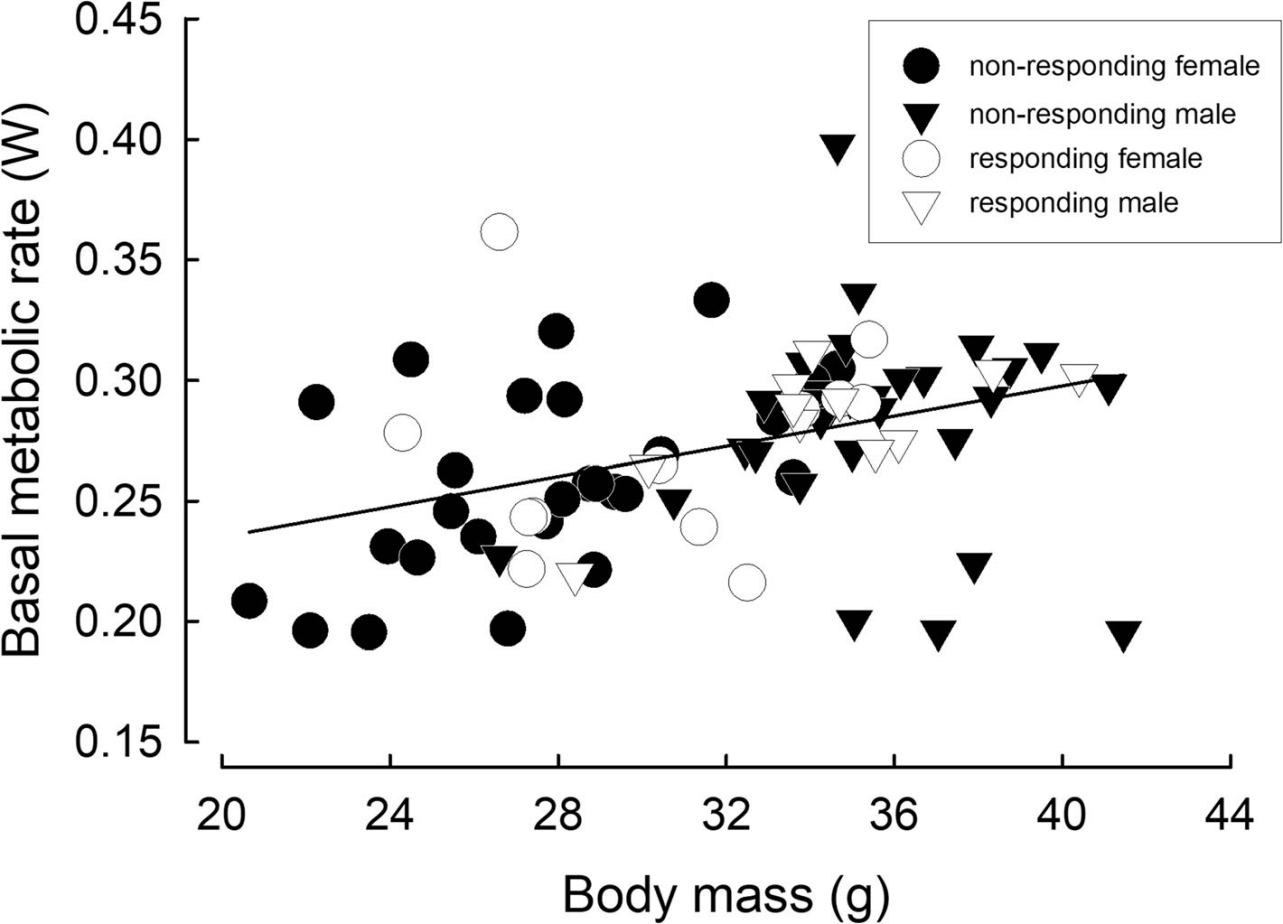
Relationship between basal metabolic rate and body mass (y = 0.003 x + 0.17, r^2^ = 0.14, F_(1, 79)_ = 13.38, *P* < 0.001) in responding and non-responding Siberian hamsters

Mean parental BMR equaled 0.27 ± 0.01 W and correlated with hamster *m*_b_ (GLM: F_(1, 81)_ = 6.59, *P* = 0.012). After adjusting for *m*_b_, BMR did not differ between reproducing and non-reproducing individuals (GLM: F_(1, 81)_ = 2.17, *P* = 0.14), between sexes (GLM: F_(1, 81)_ = 0.01, *P* = 0.93) or between phenotypes (GLM: F_(1, 81)_ = 0.19, *P* = 0.66; Fig. 3, Table 3).

**Table 3:** Comparison of basal metabolic rate and oxidative status between winter phenotypes of Siberian hamster

		Phenotype	
Life history trait		responding	non-responding
Basal metabolic rate (W)		268 ± 0.009	273 ± 0.005
Reactive Oxygen Metabolites (mg dL^− 1^)	male	15.50 ± 8.00	9.71 ± 1.54 ^a^
	female	10.77 ± 1.88 ^b^	9.67 ± 2.91
Biological Antioxidant Potential (μM Vit-C L^− 1^)	male	3525.98 ± 360.47	3596.77 ± 237.59
	female	3517.56 ± 655.83 ^b^	3230.47 ± 608.63 ^b^

Values are mean ± SD or marginal mean ± SE in case of Basal metabolic rate

^a^ difference between reproductive status; *P* < 0.05

^b^ difference between sexes within phenotype; *P* < 0.05

**Fig. 4:**
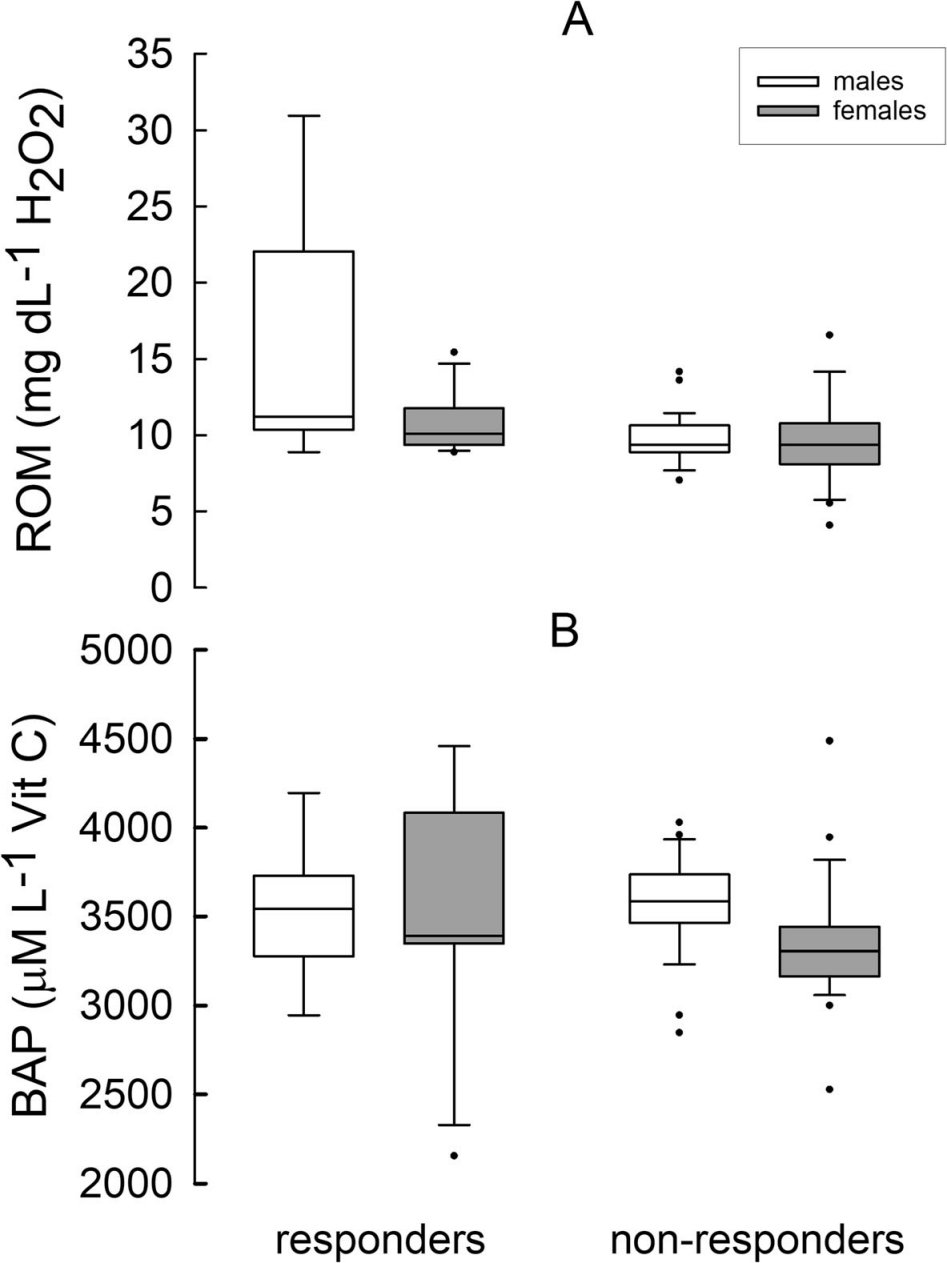
**a**: Reactive oxygen metabolites (ROM) and **b**: biological antioxidant potential (BAP) in plasma in responding and non-responding Siberian hamsters. Boxes cover the 25th to 75th percentiles, lines inside boxes indicate median, while whiskers indicate maximum value below upper fence and minimum value above lower fence. Dots indicate outliers

Concentration of ROM did not differ between reproducing and non-reproducing hamsters (GLM: F_(1, 70)_ = 0.36, *P* = 0.55), but it differed between phenotypes (GLM: F_(1, 70)_ = 6.80, *P* = 0.01) and sexes (GLM: F_(1, 70)_ = 6.60, *P* = 0.01; Fig. 4 a). Responders had higher concentration of ROM than non-responders and in males it was higher than in females. These differences most probably resulted from high concentration of ROM in responding males (GLM: F_(1, 70)_ = 6.24, *P* = 0.01; Table 3). Biological antioxidant potential did not differ between hamsters of different reproductive status (GLM: F_(1, 70)_ = 1.37, *P* = 0.25) or between phenotypes (GLM: F_(1, 70)_ = 0.01, *P* = 0.96; Fig. 4 b). However, there was an effect of sex on BAP (GLM: F_(1, 70)_ = 5.32, *P* = 0.02), and in males of both phenotypes BAP was higher than in females (Table 3).

## Discussion

In small mammals of the Temperate-Zone seasonal adjustments, including gonadal regression, molting to winter coat and heterothermy reduce energy expenditure in winter. Previous studies suggested that individuals which respond to winter photoperiod may have longer life span, and higher reproductive success than non-responding ones [17, 21, 25, 55]. Here we aimed to test the hypothesis that different strategies of surviving winter are related to differences in life history traits. To do so, we compared reproductive characteristics of two phenotypes of Siberian hamsters – responding and non-responding to seasonal changes in day length. We also analyzed the relationship between reproduction, energy metabolism, and oxidative status in this species.

### Effect of phenotype on life history traits

Out of the studied population we created 11 pairs of responding and 29 pairs of non-responding hamsters. All pairs of responders bred successfully, while among non-responders 17 pairs did not breed at all. A possible explanation for that could be different rate of aging in both phenotypes. Previous studies which showed delayed reproductive ageing in individuals maintained under short photoperiod provide indirect support for this hypothesis [25, 26]. Place and Cruickshank [25] suggested that non-responders age faster than responders and found that female Siberian hamsters responding to short days had greater number of ovarian primordial follicles at older age than non-responding ones. Our animals were about one year old when paired. In a closely related species, the Djungarian hamster *Phodopus campbelli*, symptoms of reproductive aging occured already in 6-month old animals, and in 8-month-olds fertility (delivery success) and fecundity (litter size and weaning success) were reduced by half [56]. Thus, it is possible that our non-responding individuals were reproductively older than responding ones. One could argue that another reason for not breeding of non-responders was low *m*_b_ of non-breeding females. They were ~ 15% lighter than the breeding ones and after winter-like acclimation they increased *m*_b_ slower than breeding females (Table 2). However, this explanation seems unlikely because at the same time some of breeding females were as small as non-breeding ones.

We found a clear correlation between photoresponsiveness and time to first reproduction. Individuals of the photo-responding phenotype commenced breeding about two weeks later than non-responding ones (Table 1). Arguably, delayed breeding in responders resulted from gonadal regression in this phenotype [8, 57, 58]. Likewise, in some hibernating species, e.g. golden-mantled ground squirrels *Callospermophilus lateralis* and Richardson’s ground squirrels *Urocitellus richardsonii*, males terminate hibernation even a month before females to rebuild gonads and *m*_b_ prior to breeding season [59, 60]. In responding Siberian hamsters time of commencement of breeding correlated negatively with sire body mass – smaller responding males required more time to achieve reproductive activity (Fig. 2). Although hamsters may mate on the day of parturition, the second litter is usually delayed [61]. Here, independent of phenotype, most animals delivered second litter within ~ 32 days after delivery of the first one. There was also no difference between responders and non-responders in the litter size (Table 1). In both phenotypes it was ~ 5 pups, that is within the typical range for Siberian hamsters [54, 62, 63]. In the same species Place an co-authors [26] found that dams which responded to short photoperiod produced bigger litters than non-responding ones, but in that study non-responsiveness was induced by pinealectomy. In our study, independent of the parental phenotype, the growth rate of pups was ~ 0.7 g per day which corresponds to a range reported previously for this species (0.56–0.94 g day^− 1^, depending on ambient temperature and litter size) [54, 61–63]. Because males were in the nests only with the first litters, and the growth rates were similar in both litters, these results support previous findings that in Siberian hamsters presence of a sire does not affect offspring growth rate or pup survival [53].

Despite similar litter size and offspring growth rate, responders delivered smaller pups than non-responders, and this difference was maintained until weaning (Table 1). Offspring mass increased with subsequent litters (Fig. 1a-c), most probably as a result of increasing *m*_b_ of dams, which were 15% heavier at the end of reproduction than just before the start of breeding. In Djungarian hamsters offspring mass increased with dam age, whereas litter size decreased [56]. Positive relationship between maternal size and offspring size (MSOS) is common among species, from invertebrates to vertebrates [64, 65]. The source of MSOS correlation is still debatable, however it is possibly related to maternal age, nutritional status and body condition [66]. Most data for the positive effect of sire *m*_b_ and paternal care on offspring condition come from avian studies [67–69], but there is also strong evidence for Djungarian hamsters [53, 54, 61, 63, 70–72]. Conversely, in Siberian hamsters the presence of male in the nest is not necessary and does not affect pup survival and development [53].

In individually kept hamsters there were no differences between males and females in nest building behavior (Table 1). During summer acclimation number of animals that built a nest from paper tube supplied to the cage, or the degree of chewing it, did not differ between sexes or phenotypes. However, after acclimation to winter-like conditions among responders more individuals built nests and chew more paper tubes than among non-responders. Apparently, this correlated with smaller *m*_b_ of responders, who presumably used their nest to increase thermal insulation.

### BMR and oxidative status

Investment into reproduction requires significant increase in metabolism [38, 73, 74]. Female laboratory Swiss mice selected for high BMR produced more milk and presented better parental care, measured as the offspring growth rate, than mice with low BMR [75, 76], whereas eastern chipmunks *Tamias striatus* with higher daily energy expenditure produced bigger litters [74]. However, in C57 mice [77] and in Siberian hamsters (this study) breeding and non-breeding individuals did not differ in BMR. Similarly, there was no difference between responders and non-responders (Table 3). One could argue that this lack of difference was an effect of measuring metabolism even a month after weaning of the last litter. However, McLean and Speakman [78] found that elevated metabolism in female brown long-eared bats *Plecotus auritus* was maintained even 80 days after weaning. Thus, we argue that Siberian hamsters did not increase BMR during reproduction. It agrees with the results showing that reproduction is not demanding for animals in natural environments [42, 44, 79, 80]. In many species dams adjust litter size or offspring mass to their current parental efficiency, therefore only forced increase of reproduction costs (e.g. enlarged litter size) would lead to increased metabolism [81–83].

The theory of increased susceptibility to oxidative stress during pregnancy and lactation has a long history [45, 84], however its experimental support is ambiguous. Increase of metabolism and ROM generation not necessarily result in oxidative stress [40, 46]. In reproducing laboratory mice oxidative damage increased in plasma [49] but decreased in liver [38, 49]. In bank voles *Myodes glareolus* reproduction led even to decrease of oxidative damage to lipids but did not change the oxidative damage to proteins [46]. Moreover, oxidative stress increased with litter size in mice [38] and eastern chipmunks [74], but not in bank voles [46] or canaries *Serinus canaria* [40]. Relationship between antioxidant capacity and reproduction is also equivocal. Reproducing mice showed lower [85], higher [38], or unchanged [48] activity of antioxidant enzymes. Antioxidant capacity increased with number of nestlings in male zebra finches *Taeniopygia guttata,* but decreased in females [41], decreased in both sexes of great tits *Parus major* [42], but did not change in striped hamsters *Cricetulus barabensis* [43].

Oxidative stress may also be understood as a proxy for somatic maintenance – increasing when resources available for somatic maintenance are insufficient [37, 40]. Based on our initial prediction that responders and non-responders differ in life history traits, we expected differences in their oxidative status after reproduction [37, 86]. However, we neither found differences between phenotypes in ROM concentration nor in BAP (Table 3, Fig. 4). We also did not find differences between breeding and non-breeding individuals. Under limited resource availability reproduction comes at a cost of self-maintenance, what may result in lower investment in antioxidant defense and eventually in oxidative stress [33–36, 39, 86, 87]. According to the theory of ageing, the loss of cell function during senescence is triggered by a shift in the redox state of the cell and oxidative damage induced by free radicals [88, 89]. This could result in accumulating deleterious mutations and shortening life span [88, 90]. Indeed, negative relationship between investment in reproduction and longevity was well established ([91–95], but see [96, 97]). In contrast, heterothermy use was considered to extend life span [17, 18, 20–23] and to reduce the extinction risk [19]. Moreover, even a small decrease in body temperature may lead to lower oxidative stress and may increase life span, as in transgenic mice with normothermic body temperature lower by 0.5 °C than in wild-type C57/BL6 mice [24]. Siberian hamsters kept under short days not only enter daily torpor but also decrease their normothermic body temperature by ~ 0.7 °C [98]. However, we found no differences in oxidative status between individuals which were using daily torpor during acclimation to winter-like conditions (responders) and those which were not (non-responders). All individuals had similar ROM and BAP levels, apart from responding males which had ~ 50% higher ROM concentration (Table 3). On the one hand this could result from the higher growth rate after winter-like acclimation in responders than in non-responders, but on the other hand it suggests impaired somatic maintenance in post-reproductive period. Higher ROM concentration only in responding males suggests that their antioxidant defense was less effective than in females, which could have been protected by antioxidant properties of estrogens [99, 100]. Yet, overall, males had higher BAP level than females (Fig. 4), suggesting that despite higher ROM concentration, responding males were not subjected to oxidative stress during reproduction.

## Conclusions

To the best of our knowledge this study is the first attempt to compare the reproductive characteristics of phenotypes of the same species which differ in their response to short photoperiod. On the one hand, cessation of reproduction in winter and *m*_b_ reduction in responding animals delayed commencement of breeding and resulted in smaller offspring *m*_b_. On the other hand, the number of non-breeding non-responders suggests that they aged faster than responders. Despite above differences in life history characteristics, both phenotypes did not differ in metabolic rate and oxidative status, suggesting no effect of reproduction on future investment in somatic maintenance. Delayed breeding in responding pairs and high post-reproductive ROM concentration in male responders support our hypothesis that differences in the adjustment to winter conditions result in different characteristics of life history traits. Because litter size and growth rate were the same in responders and non-responders, and assuming the same resource availability for both phenotypes, we propose that despite differences in the strategy of managing energy resources in winter, they achieve similar fitness, what may explain coexistence of both phenotypes in the population.

However, here we compared only two extreme phenotypes, responding and non-responding to short photoperiod, whereas within one population of Siberian hamsters we can observe entire spectrum of winter phenotypes [101]. Such polymorphism may be beneficial in stochastic environments, where environmental conditions may differ between winters. We propose that non-responding phenotype may be particularly beneficial during mild winters, in the environment with abundant energy supplies and immediate access to food after winter. Responders would be favored during harsh winters, when energy savings mechanisms (e.g. decrease in *m*_b_, gonadal regression and daily torpor) are the most important phenotypic adjustments. If so, from an evolutionary point of view, none of the phenotypes would be impaired when compared to another and polymorphism would be maintained in population.

## Methods

All experimental procedures were approved by the Local Committee for Ethics in Animal Research in Bydgoszcz, Poland (decisions nos. 3/2015, 31–33/2015, 35/2015).

### Animals and housing

We used Siberian hamsters from the outbred colony maintained at the Department of Biology and Environmental Protection at Nicolaus Copernicus University in Toruń, Poland. After birth animals were kept under summer-like conditions, i.e. at ambient temperature (*T*_a_) = 18 ± 2 °C and long photoperiod (16 L:8 D) for 4 months. Then, to induce change to winter phenotype, animals were transferred to winter-like conditions (*T*_a_ = 10 ± 2 °C and short photoperiod (8 L:16 D)) for 3 months. After completing the acclimation to winter-like conditions hamsters were transferred to summer-like conditions again and 2 weeks later they were paired for breeding. Animals were fed with standard rodent food (Labofeed, Morawski, Kcynia, Poland) and supplied with drinking water ad libitum.

### Defining winter phenotypes

Winter phenotypes were determined based on fur coloration and use of torpor. A hamster was classified as responding if it at least partially changed fur color and entered at least one episode of torpor (subcutaneous temperature; *T*_sc_ ≤ 32 °C), and as non-responding if it remained grey and did not enter torpor throughout winter. Animals that showed only one trait of winter phenotype (i.e. only white fur or only torpor) were classified as partial responders [101] and were excluded from analyses. To assess the use of torpor, we monitored individuals’ *T*_sc_ with miniature data loggers. Loggers (model TL3–1-27; accuracy of 0.3 °C between 0 °C and 45 °C, constructed by Dr. Dmitry Petrovski, Russian Academy of Sciences, Novosibirsk, Russia) weighted ~ 0.8 g and allowed downloading data wirelessly. Before implantation, loggers were pre-calibrated against a precise mercury-in-glass thermometer between 15 °C and 40 °C. After ~ 6 weeks of winter acclimation loggers were implanted subcutaneously into the interscapular region under ketamine (40 mg kg^− 1^; Ketamina 10%, Biowet, Puławy, Poland) and xylazine (8 mg kg^− 1^; Sedazin 2%, Biowet, Puławy, Poland) anesthesia. The incisions were closed with absorbable sutures (Safil 5/0, Aesculap AG, Tuttlingen, Germany). After surgery all hamsters were kept in the laboratory for 1 day in individual cages (under short photoperiod but *T*_a_ = 22 °C) and then were transferred back to the animal facility room. Subcutaneous temperature was recorded every 10 min and downloaded every 2 weeks.

### Life history traits

Two weeks after changing conditions to summer-like hamsters (~ 1 year old) were paired into 40 phenotypically-matching pairs composed of responding or non-responding hamsters (11 pairs of responders and 29 pairs of non-responders). All animals were weighed before being paired and also after birth of the second litter (sires) or after weaning of the second litter (dams). Animals remained paired for 14 weeks or until delivery of the second litter. Immediately after birth of the second litter, males were separated from females to prevent further breeding. Eventually, 12 pairs of non-responding hamsters and 11 pairs of responding hamsters bred successfully and delivered at least one litter. Seventeen out of non-responding pairs did not breed at all and these animals were used as a control group in the analyses of the cost of reproduction. Then we determined life history traits associated with reproduction: time of commencement of breeding, time interval between consecutive litters, litter size, mass of litter at 3rd, 10th and 18th day of life and growth rate of offspring. Whole litters were weighed together, to the nearest 0.1 g (SPU402, OHAUS, Parsippany, NJ, USA) and mean *m*_b_ of individual offspring was calculated as total mass of the litter divided by number of offspring.

### Nest-building behavior

During acclimation to summer- and winter-like conditions, before mating, nest-building behavior tests were done in individual hamsters twice in each season (10 days apart). Toilet paper tubes were used as a nesting material. Paper tubes (~ 5.50 g, ~ 10 cm length and ~ 3 cm in diameter) were dried for 72 h at 50 °C, weighed, and put into the animal’s home cage. After 48 h, non-chewed remains of tubes were collected, dried for 72 h, and weighed to calculate the proportion of tube chewed by a hamster. In addition, we analyzed the propensity of animals to build a nest. We did not assess the complexity of nest structure but considered any roundish pile of paper pieces with a visible print of animal’s body as a nest.

### Oxidative stress and antioxidant capacity

At least 2 weeks, but not later than 6 weeks after weaning of the second litter we measured OS and AC in plasma. In non-reproducing pairs oxidative status was assessed at the same time as in reproducing ones. OS and AC were measured using Free Radical Analytical System (FRAS4 evolvo, H&D, Parma, Italy; henceforth: H&D). This system measured the concentration of reactive oxygen metabolites (ROM) and biological antioxidant potential (BAP) in plasma. ROM concentration was measured with dROM-kit (d-ROM-kit, REDOX Kit; H&D) as a level of total H_2_O_2_, and BAP was measured using PAT-kit (Plasma Antioxidant Test, REDOX Kit; H&D) as a vitamin C concentration. Blood sample (~ 100 μl) was taken from the retro-orbital sinus by capillary puncture. Immediately after bleeding we applied analgesic eye drops into hamster’s eye (Alcaine 5 mg/ml, Alcon Polska, Warsaw, Poland).

### Basal metabolic rate

Basal metabolic rate was measured at least 2 weeks, but not later than 4 weeks, after weaning of the last litter or after separation of control pairs by indirect calorimetry using an open-flow respirometry system (Sable Systems International, Las Vegas NV, USA; henceforth: SSI). Gas exchange was measured for ~ 8 h within the thermoneutral zone of Siberian hamsters (at 29 ± 1 °C [102, 103]) and BMR was calculated as the lowest rate of O_2_ consumption during 3 min in two last hours of the test. All data were acquired using ExpeData software (SSI) at 0.5 Hz. During measurement animals were sealed in 0.85 L respirometry chambers made of transparent polypropylene food containers (HPL 808, Lock&Lock, Hana Cobi, South Korea) which were placed in a temperature controlled cabinet (ST-1200, Pol-Eko-Aparatura, Wodzisław Śląski, Poland). Because BMR measurement requires post-absorptive conditions, hamsters did not have access to water and food during the measurement. Previously we found that this period was sufficient to ensure post-absorptive conditions in the species [103]. We used two parallel respirometry systems, which allowed to simultaneously measure 14 individuals.

Air was pulled from outside the building using air pump (5HCE-10-M553, Gast Manufacturing, Benton Harbor, MI, USA) and compressed in a balloon. Then air was dried and scrubbed of CO_2_ with a PureGas Generator (Puregas, Westminster, CO, USA). The main air stream was then split into chambers and a reference gas stream. Air flow rate (~ 330 mL min^− 1^) was regulated upstream of each respirometry chamber with a precise needle valve. Air streams leaving the respirometry chambers were selected sequentially with a computer-controlled multiplexer (Intelligent Multiplexer V3, SSI) and flow rate was measured downstream with a mass flow meter (FlowBar-4, SSI). After flow measurement, air stream was subsampled at a rate of ~ 100 mL min^− 1^ and water vapor pressure was measured (RH-300, SSI., USA). Then air stream was pulled through a nafion dryer tubes (product number 17049, VacuMed, Ventura, CA, USA) embedded in silica gel and finally was dried with magnesium perchlorate (product number 11636.36, VWR International, Gdańsk, Poland). Then concentrations of O_2_ and CO_2_ were measured. Oxygen consumption rate $\left(\dot{V}{O}_2\right)$ and CO_2_ production rate $\left(\dot{V}{CO}_2\right)$ of seven individuals were measured with FoxBox-C integrated CO_2_ and O_2_ analyzer (SSI). In the remaining seven hamsters we used FC10a analyzer (SSI) and CA10 analyzer (SSI) to measure $\dot{V}{O}_2$ and $\dot{V}{CO}_2$, respectively. Gas exchange of each animal was recorded for 5 min, every 44 min and the baseline gas concentration readings were done every 20 min. Metabolic rate was calculated after Lighton and coauthors [104] as follows:

$$ MR\ (W)=\frac{\dot{V}{O}_2\left(16+5.164\times RER\right)}{60} $$

where $\dot{V}{O}_2$ is the rate of oxygen consumption (ml O_2_/min) and $RER=\frac{\dot{V}{CO}_2}{\dot{V}{O}_2}$ was calculated from recorded $\dot{V}C{O}_2$ and $\dot{V}{O}_2$. All individuals, regardless of phenotype and sex, had similar RER ranging between 0.79 ± 0.03 in summer and 0.83 ± 0.05 in winter.

### Statistical analysis

We divided animals into two groups reflecting reproductive status: breeding and non-breeding animals. Animals in the latter group were paired but for unknown reasons did not breed. Only non-responding individuals did not breed. To analyze whether phenotype and reproductive status affect parental *m*_b_ before and after breeding, we used linear mixed model (LME) with animal ID as a random factor, phenotype, sex, reproductive status and time of measurement (before or after breeding) as fixed factors and two interactions: sex × reproductive status and phenotype × time of measurement.

To analyze the effect of phenotype on litter size and offspring *m*_b_, we used LME with pair ID set as a random factor. Consecutive litter number and parental phenotype were used as fixed factors. Additionally, in the model analyzing offspring *m*_b_ we used litter size and day of measurement (3rd, 10th or 18th) as a covariates. We used the day of *m*_b_ measurement as a continuous variable, to obtain information about the body mass gain (growth rate). Model included also two interactions: parental phenotype × day of measurement and parental phenotype × litter size. We did not include dam and sire *m*_b_ in the analysis because of its non-significant effect on litter size (dam: F_(1,19.17)_ = 0.03, *P* = 0.87; sire: F_(1,19.28)_ = 0.10, *P* = 0.76) and offspring *m*_b_ (dam: F_(1,18.26)_ = 0.46, *P* = 0.50; sire: F_(1,17.99)_ = 0.80, *P* = 0.38).

Because of small sample size and non-normal distribution of data, effect of phenotype on time of commencement of breeding and time interval between consecutive litters were analyzed by Mann-Whitney U test. Medians were compared by Mood’s median test. We used Pearson correlation to relate time of commencement of breeding and time interval between consecutive litters with *m*_b_ of dams and sires, separately for responders and non-responders.

To analyze the effect of reproduction and phenotype on BMR, ROM concentration and BAP we used General Linear Model (GLM) with phenotype, sex, and reproductive status as fixed factors. In the model analyzing the variability of BMR we used *m*_b_ as covariate. The initial models included also interaction of sex and phenotype, but finally, this interaction was excluded, except for the model analyzing the variability of ROM concentration.

Repeatability of the proportion of paper tube chewed by individual was calculated with LME, and repeatability of propensity to build the nest was calculated with Generalized Linear Mixed Model (GLMM) and binary probit model. To test if phenotype and breeding status affect nest-building behavior we used LME with ID as a random factor, sex, phenotype, breeding status, season and trial as fixed factors, and interactions: phenotype × season and season × trial. To test if phenotypes differ in ability to build the nest we used contingency table using Poisson errors. We used four explanatory variables: phenotype, sex, season and breeding status.

All results, except for time of commencement to breeding and time interval between consecutive litters (median ± SE), are presented as mean ± SD. Statistical significance was accepted at *P* < 0.05.

## Abbreviations

AC: Antioxidant capacity
BAP: Biological antioxidant potential
BMR: Basal metabolic rate
*m*_b_: body mass
OS: Oxidative stress
ROM: Reactive oxygen metabolites
